# Participants’ reactions to the inclusion of sexual orientation and gender identity measures in an established large-scale cohort: the California Teachers Study

**DOI:** 10.1093/aje/kwaf074

**Published:** 2025-04-16

**Authors:** Kristen E. Savage, Christopher W. Wheldon, Emma S. Spielfogel, Brittany M. Charlton, Caroline A. Thompson, Christine N. Duffy, Maria Elena Martinez, James V. Lacey

**Affiliations:** 1 Department of Computational and Quantitative Medicine, Beckman Research Institute, City of Hope, Duarte, CA 91010, United States; 2 Department of Social and Behavioral Sciences, College of Public Health, Temple University, Philadelphia, PA 19122, United States; 3 Department of Population Medicine, Harvard Medical School and Harvard Pilgrim Health Care Institute, Boston, MA 02215, United States; 4 Department of Epidemiology, Harvard T.H. Chan School of Public Health, Boston, MA 02115, United States; 5 Department of Epidemiology, University of North Carolina, Chapel Hill, NC 27599, United States; 6 Department of Epidemiology and Biostatistics, University of California San Francisco, San Francisco, CA 94143, United States; 7 Herbert Wertheim School of Public Health and Human Longevity Science, University of California at San Diego, La Jolla, CA 92093, United States; 8 Moores Cancer Center, University of California at San Diego, La Jolla, CA 92093, United States

**Keywords:** sexual orientation, gender identity, retention, sexual and gender minority populations, longitudinal study

## Abstract

Sexual and gender minority (SGM) populations are more likely than non-SGM populations to experience poor health outcomes but are underrepresented in research. The California Teachers Study is a large prospective observational cohort that, in its 2017-2019 survey, included sexual orientation and gender identity (SOGI) measures and also asked participants to provide feedback on the questionnaire. We conducted an inductive content analysis of participants’ feedback responses and used these qualitative codes to (1) identify which participants commented on SOGI and (2) establish whether these participants had a positive, negative, or ambiguous reaction to the measures. We used chi-square tests and multivariable logistic regression to evaluate whether demographic factors were associated with reaction type. A total of 373 (2%) of 19 496 respondents commented on the SOGI questions. Of these, 41% had negative reactions, 35% had ambiguous reactions, and 25% had positive reactions. Younger age (<65 years) was positively associated with positive reaction (odds ratio [OR] = 2.30; 95% CI, 1.23-4.32). Non-White participants had lower adjusted odds of having a negative reaction to the SOGI measures compared to White participants (OR = 0.38; 95% CI, 0.17-0.87). In this large established study of older adults, feedback on SOGI measures was rare but differed in frequency and content by participants’ demographics.

## Introduction

Sexual and gender minority (SGM) populations are more likely to experience poorer physical and mental health outcomes compared with non-SGM persons,^1-6^ but understanding of the causes of these disparities is limited by inadequate, and often nonexistent, data. Data are also lacking on vulnerable SGM subpopulations, such as older SGM people at risk for chronic conditions like cancer. Estimates suggest there will be more than 5 million SGM-identifying adults age 50 and older in the United States by 2060.^7^ However, SGM populations, and older SGM subpopulations specifically, are still underrepresented in chronic disease research.^8^

Collecting sexual orientation and gender identity (SOGI) data in clinical research and public health surveillance is a national priority.^9,10^ Both the National Cancer Institute and the National Institutes of Health (NIH) have established SGM research as an area of institutional import, and SGM populations are now an NIH-designated health disparity population.^11-13^ Guidelines issued by the National Academies of Sciences, Engineering, and Medicine recommend that measures of sex, gender, and sexual orientation be included on federal surveys as demographic measures similar to race and ethnicity, and there is strong support for a 2-step approach to measuring assigned sex at birth and current gender identity.^9^ Expanding SOGI data collection efforts, including among older adults, is key to reducing disparities and increasing equity for SGM populations.^10^

Cancer epidemiologic cohorts (CECs)—large-scale, long-term studies that enroll a single group of people at baseline and then follow those individuals over time, often for decades—are particularly well equipped to advance research on SGM communities. The wealth of longitudinal, individual-level phenotype and outcome data collected in CECs could enable researchers to study risk factors and outcomes, with 1 caveat: most CECs have not collected SOGI data from their participants.^14-16^

Public perceptions about and understanding of SOGI have shifted drastically since the 1990s and early 2000s, when many CECs were established. In 1996 when the California Teachers Study (CTS) completed enrollment, 40% of US adults surveyed by Gallup’s public opinion poll said that gay and lesbian people should not be hired as elementary school teachers; 34% thought gay and lesbian people should not be high school teachers.^17^ In 2019, those numbers had dropped to 18% and 15%, respectively.^17^ While social norms and laws about SOGI have changed over the life course of these CECs, research priorities about SGM populations have also transformed.^17,18^

As CECs now consider how to contribute to SGM research, questions emerge about whether participant reactions to SOGI measures will affect cohort retention; high retention is essential to CECs’ validity.^19,20^ Findings from multiple clinical and patient-care settings suggest that both SGM and non-SGM individuals are willing to answer SOGI questions within a healthcare setting,^1,21,22^ and some cohorts have successfully implemented SOGI measures in their studies,^23-25^ but there is a paucity of research on how older participants react to these measures within established CECs.

When the CTS developed its sixth study-wide questionnaire (Q6) in 2016, we wondered whether including SOGI measures for the first time might offend some participants (and lead to nonresponse or study withdrawal) or delight some participants (and increase engagement). Q6 was also the first web-based survey (Q6web) offered to study participants. To capture feedback on the questionnaire experience, Q6web included 3 optional open-ended questions for respondents to provide comments. In their written responses, CTS participants offered both general questionnaire feedback and measure-specific commentary, including on the SOGI questions.^26^ The purpose of this study was to (1) describe CTS Q6web respondents’ reactions to the inclusion of SOGI measures as stated in their feedback comments, (2) characterize the demographics of Q6web respondents who reacted to the inclusion of the SOGI measures, and (3) assess the potential for CTS participant attrition due to the inclusion of SOGI measures in this long-standing cohort.

## Methods

### Study setting and population

The CTS began when 133 477 female members of the California State Retirement System (CalSTRS), mostly teachers and administrators, agreed to enroll by completing a 16-page questionnaire (Q1, 1995-1996) that collected information on health, family history, diet, physical activity, reproductive history, and other exposures.^27^ Since baseline, additional exposure, lifestyle, and self-reported health data have been collected through 5 follow-up questionnaires: Q2 (1997-1998), Q3 (2000-2002), Q4 (2005-2008), Q5 (2012-2015), and Q6 (2017-2019). Follow-up also includes annual linkages with cancer, hospitalization, and mortality data; newsletters and information disseminated via the CTS website; and additional projects (eg, in-person biospecimen collections) where participants interacted with study personnel.^28^

This study was approved by the Institutional Review Boards of City of Hope and Temple University. CTS participants provided informed consent at study enrollment and when they completed Q6. As in all CTS surveys, participants could skip any questions they did not want to answer.

### CTS questionnaires

The first 5 questionnaires (Q1-Q5) used scannable surveys sent via mail. Q6web was available online to study participants who had provided email addresses. Participants who did not complete Q6web or had not provided an email address received a scannable paper version sent via mail (Q6paper), which had identical scientific content to the online version, as previously described.^29^

### Q6 content, including SOGI

Copies of all CTS questionnaires, including Q6, are available on the CTS website.^30^ Q6 updated previously collected data (eg, physical activity, family history, medication use) and introduced new topics, such as financial stress, medicinal cannabis, and SOGI.^26^

For SOGI, participants were asked to answer a single question about sexual orientation and 2 questions to assess sex and gender identity^31^: “Which of the following best represents how you think of yourself?” with 6 response options (*straight*, *lesbian or gay*, *bisexual*, *other*, *don’t know/not sure*, and *prefer not to answer*); “Which sex were you assigned at birth?” with 4 response options (*female*, *male*, *intersex*, and *prefer not to answer*); and “Please mark the gender identity that best describes you currently” with 6 response options (*female*, *male*, *transgender*, *intersex*, *other*, and *prefer not to answer*).

Because Q6web was the first CTS survey administered online, we included 3 optional open-ended questions about the questionnaire as a whole: “What did you like best about this questionnaire?”; “How could we improve this questionnaire?”; and “Is there anything else you would like us to know?”

### Analytic population and data

This analysis includes the *n* = 19 498 participants who completed Q6web and had the opportunity to answer the 3 feedback questions. We excluded 1 participant who asked that her data only be used for breast cancer research. We excluded 1 participant who responded that he was assigned male at birth and identified as male today (cisgender male). At study enrollment (1995-1996), CTS participation was limited to participants who identified as female; therefore, this cisgender male respondent was considered a survey error. Age was based on self-reported date of birth from Q1; race/ethnicity was also collected in Q1. Marital status, household income, and retirement status were collected in Q6.

### Coding

#### Qualitative coding

Of the 19 498 participants who completed Q6web, 11 407 (59%) responded to at least 1 feedback question. As Q6web was being completed, coder A (K.E.S.) reviewed all 11 407 participants’ responses to the 3 feedback questions for the purpose of understanding participants’ experiences and identifying any comments that required immediate follow-up. Soon after that review began, it expanded to an inductive content analysis and the reviewer assigned qualitative codes to all participants’ comments.^32,33^ Coder A completed that analysis within the questionnaire software (Qualtrics, Salt Lake City, UT) from 2017 to 2019.^34^

In 2022, coder A re-reviewed all the original qualitative codes and identified 13 codes that were directly or peripherally related to the SOGI measures; 405 questionnaire respondents had 1 or more of these codes. Coder A developed a codebook that defined all possible qualitative codes relevant to the SOGI measures and added descriptions of how each code had been used in 2017-2019 (Appendix S1).

#### Double coding

**Figure 1.**
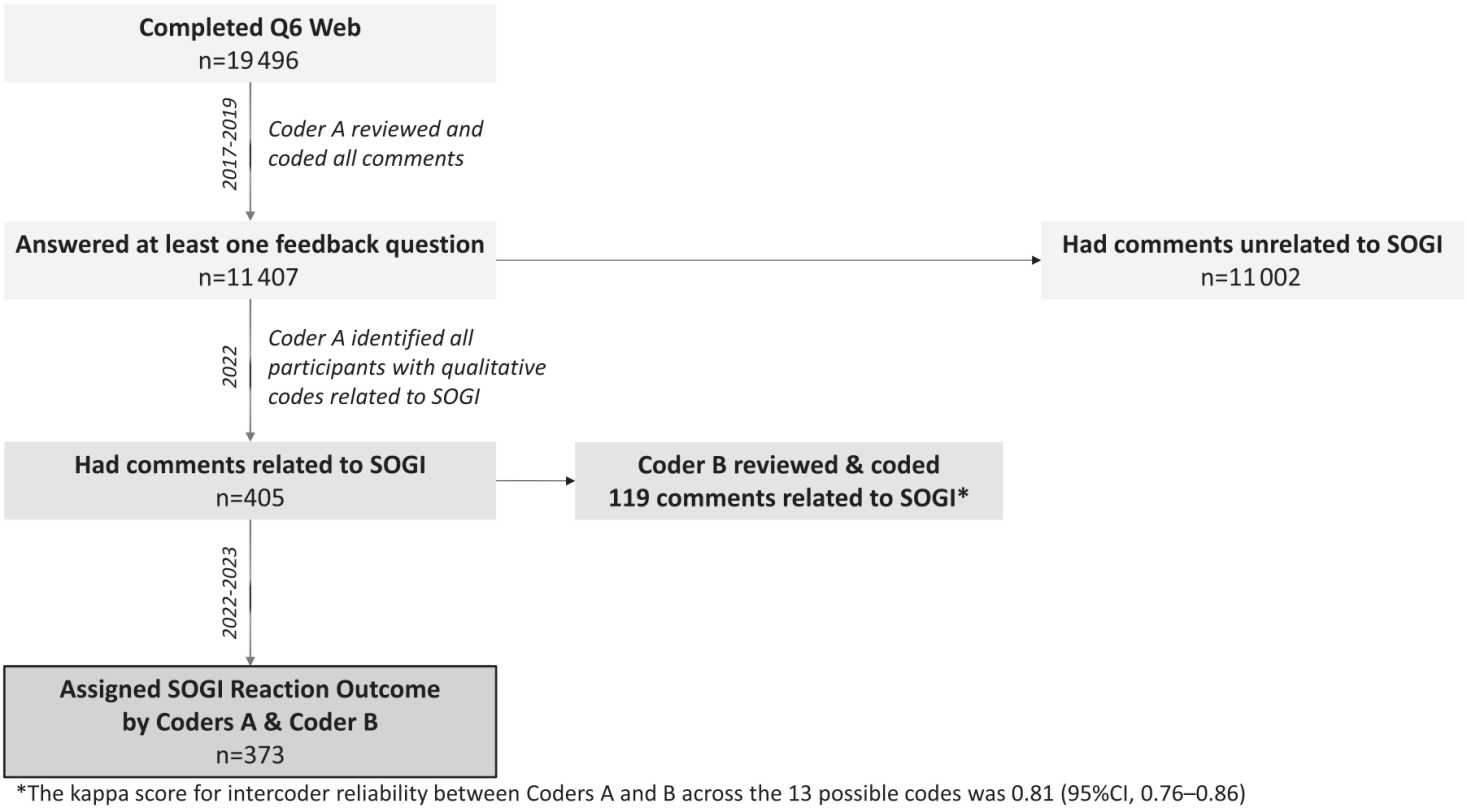
Qualitative and quantitative coding process of participant comments on Q6.

Coders A and B (C.W.W.) reviewed the codebook together. Coder B then used QDA Data Miner Lite (Provalis Research, Montreal, Canada) to assign qualitative codes to comments from a random sample of the 405 participants whom coder A had identified as having feedback relevant to SOGI.^35^ After coder B assigned qualitative codes to 119 of the 405 participants (29%), we calculated a kappa score to establish concordance between coders A and B (Figure 1). The kappa score for intercoder reliability between coders A and B across the 13 possible codes was 0.81 (95% CI, 0.76-0.86). Of the 13 qualitative codes described in the codebook, 4 were unrelated to SOGI (ie, some of these participants also commented on non-SOGI items). When restricted to the positive and negative comments related to the SOGI measures, the intercoder reliability was 0.89 (95% CI, 0.82-0.95). Based on the kappa scores, we decided to stop coder B’s review and use coder A’s codes for this analysis.

#### Quantitative measures

Coders A and B then collaboratively identified 9 of the 13 original qualitative codes as appropriate for characterizing SOGI reactions (Table 1). Four qualitative codes (“remove marijuana questions,” “remove income question,” “remove organic food questions,” and “full study refusal”) were excluded from the analysis.

Together, the 2 coders established criteria to assign each of the 9 qualitative codes to 1 of 3 SOGI reaction categories: negative, ambiguous, or positive. Codes were assigned to the positive or negative reaction groups, respectively, when they met 2 criteria: (1) expressed a positive or negative reaction that was (2) clearly related to the SOGI measures. The remaining codes were designated as “ambiguous”: the comments in this category either (1) mentioned SOGI but did not clearly express like or dislike, or (2) expressed nonspecific positive or negative statements that could not be clearly attributed to the SOGI questions. For example, “It’s pretty inclusive” (code: “inclusive”; reaction: “ambiguous”) and “This Questionnaire was much too personal” (code: “too intrusive”; reaction: “ambiguous”) include positive and negative statements, but neither comment indicates which part of the survey (eg, SOGI, medicinal cannabis, financial toxicity, etc.) was inclusive or too personal.

Using these assignment criteria, we created a single outcome variable for each participant who had commented on SOGI. Based on the codes assigned to their SOGI-related comments, participants were categorized as having either a negative, ambiguous, or positive reaction. This categorical SOGI reaction variable was mutually exclusive; participants could belong to only 1 group. Thirteen participants had comments spanning more than 1 reaction category (eg, “ambiguous and positive” or “ambiguous and negative”); these participants were assigned to the stronger reaction group (positive and negative, respectively, in the examples above). None of the participants had codes in both the negative and positive reaction groups. This allowed us to create a single quantitative SOGI reaction indicator for each participant who commented on SOGI. Of the 405 participants eligible for double coding, 373 had SOGI-related codes and were assigned a reaction outcome.

We also created a binary variable to evaluate differences between participants who had “any reaction to SOGI” (meaning a negative, ambiguous, or positive SOGI reaction) and those who did not react. Participants who either did not answer any feedback questions or provided comments unrelated to SOGI were included in the “no reaction to SOGI” group (nonreactors).

### Statistical analysis

We used chi-square tests to compare demographic differences between participants who had any reaction to the SOGI measures and those who did not, and to compare each reaction type (negative, ambiguous, positive) to nonreactors. We used logistic regression models to estimate the magnitude and directionality of the associations between the demographic factors and reacting (vs nonreacting) with ORs and 95% confidence intervals (95% CI). While we acknowledge that adjustments in descriptive epidemiology are not commonplace,^36^ we did identify several nuisance variables that represented known interdependencies among demographics in the Q6web population. For example, distributions of younger vs older participants differ by race, marital status, income, and retirement; distributions of White vs non-White participants differ by income, marital status, and retirement; and distributions of retired vs nonretired participants differ according to their income. These variables are considered to be nuisance factors in a descriptive analysis and so were adjusted to ensure that this interrelatedness did not mask the associations of interest.^36^ To avoid incorrect interpretations,^37,38^ the models are not mutually adjusted but selectively adjusted for the nuisance factors relevant to estimating the OR for each characteristic, as described in the footnotes of Tables 4-6. Sexual orientation and gender identity were not included in the models due to small cell sizes in some groups. The number of participants with missing responses to 1 or more of the covariates was small (*n* = 377, 2%); all models therefore excluded participants with any missing covariate data, and we did not impute any values for missing data. To protect confidentiality and reduce potential for identification, certain small cell sizes are suppressed in the tables. All analyses were completed using SAS version 9.4 (SAS Institute, Cary, NC) in the CTS Researcher Platform.^39^

## Results

Table 2 presents descriptive characteristics of the 19 496 participants who were eligible for this analysis. Of the 19 496 participants who completed Q6web, 19 123 (98%) did not comment on the inclusion of the SOGI measures (no reaction to SOGI) and 373 (2%) provided a comment about the inclusion of the SOGI measures (any reaction to SOGI).

Younger age and still working were positively associated with any reaction to SOGI. There was no evidence that race, marital status, or annual household income were associated with whether a participant commented on the SOGI measures.

### SOGI nonresponse

Almost all participants answered the SOGI questions. Only 351 (2%) chose to skip or select “prefer not to answer” on the sexual orientation question; 149 (1%) did so on 1 or both gender identity questions. For comparison, 3166 (16%) skipped or selected “prefer not to answer” on the income question.

### SOGI reaction type

Among the 373 respondents who had a reaction to the SOGI measures, 151 (40%) had a negative reaction, 129 (35%) had an ambiguous reaction, and 93 (25%) had a positive reaction (Table 3).

Compared to nonreactors, reactors tended to be younger on average and not retired, especially those who reacted either positively or negatively. Also compared to nonreactors, negative reactors were more often of White race, but among the no reaction, ambiguous reaction, and positive reaction groups, the percentages of White and non-White participants were similar. Additionally, negative reactors were more frequently in the group who did not respond to income questions, but no other trends by income were evident. No discernible trends in reaction were noted across groups defined by marital status (Table 3).

### Any reaction

We analyzed whether participant characteristics were associated with any reaction (ie, negative, ambiguous, or positive reaction vs no reaction; Table 4). In the bivariable model, younger age (<65) and still working (ie, not retired) were associated with having any reaction to the SOGI measures (OR = 1.40; 95% CI, 1.06-1.85; and OR = 1.36; 95% CI, 1.09-1.70, respectively). In the multivariable models, younger age was associated with reaction to the SOGI measures (OR = 1.43; 95% CI, 1.08-1.89); retirement status was not (OR = 1.24; 95% CI, 0.92-1.66).

Even after adjusting for age, race, marital status, and retirement status, “prefer not to answer” response to annual household income was positively associated with reacting to the SOGI measures (OR = 1.72; 95% CI, 1.24-2.39).

### Negative reaction

Race/ethnicity was associated with negative reactions to SOGI measures in both the bivariable and multivariable models (Table 5). Non-White participants had lower adjusted odds of having a negative reaction to the SOGI measures compared to White participants (adjusted OR = 0.38; 95% CI, 0.17-0.87).

Compared with reporting the middle household income ($100 000-$149 999), “prefer not to answer” for household income was positively associated with negative reactions to the SOGI measures (adjusted OR = 2.95; 95% CI, 1.80-4.83).

### Positive reaction

Age under 75 years was positively associated with positive reactions to the SOGI questions in both the bivariable and multivariable models (Table 6). Age groups 65-74 (adjusted OR = 1.84; 95% CI, 1.00-3.36) and younger than 65 (adjusted OR = 2.30; 95% CI, 1.23-4.32) were positively associated with positive reaction. Race, marital status, annual household income, and retirement status were not associated with having a positive SOGI reaction.

### Study attrition

Attrition was defined as participants who opted out of future CTS contact. Of the 89 participants who opted out of the CTS between October 2017 and December 2021, 4 (4%) explicitly said their refusal was due to SOGI measures, and an additional 4 (4%) referenced the questions at the end of the questionnaire as their reason for their refusal (SOGI was the last topic on the questionnaire). Of those 8 participants, 3 completed Q6web and are included in the negative reaction group described in this analysis; the other 5 completed Q6paper or declined Q6 participation.

## Discussion

Cohort studies like the CTS include participant-reported and real-world data that can help document and address SGM health disparities. Findings from this study suggest that, when given an opportunity, few participants will provide feedback about SOGI measures in CEC research; even fewer participants will react negatively to the SOGI questions; and even fewer will withdraw or opt out of future study activities because of the SOGI questions. Overall, the inclusion of SOGI measures did not appear to have a significant adverse effect on data collection.

We included the feedback questions to better understand participants’ user experience. Some participants used these questions to comment on Q6 content, specifically the SOGI measures. The differences in demographic factors associated with negative, ambiguous, and positive reactions to SOGI among Q6web respondents indicate that it is possible some participants in similar cohorts may withdraw after seeing a question they perceive as sensitive or irrelevant; however, in the CTS, those subgroups were small. Q6 was the first time participants provided open-ended feedback so we also could not assess how many participants may have withdrawn prior to Q6 because of the lack of SOGI measures in the cohort. Future research is needed to monitor attrition before and after the inclusion of SOGI items to directly assess their effect on attrition.

Nearly three-quarters (71%) of the CTS respondents were age 65+, and the study drop out was extremely low. This may be partly because perceptions about SGM populations have changed during CTS follow-up. When the CTS began, only 27% of Gallup respondents nationwide stated that marriages between same-sex couples should be recognized by the law as valid, with the same rights as “traditional” marriages; by 2017, when Q6 was disseminated, that number had grown to 64%.^17^ The positive reaction to SOGI measures among younger CTS participants may reflect this trend and the mounting belief that health research should keep pace with changing social views. Younger participants’ tendency to react positively to SOGI measures suggests including SOGI measures could help long-term retention for those younger participants. That the small subset of participants who reacted negatively to the SOGI items were in the oldest age category may also indicate that the acceptability of SOGI data collection will increase over time. Additional research in CECs could explore how potential age, period, and cohort effects influence participants’ stated beliefs about SGM populations.

Among CTS Q6web respondents, those who responded “prefer not to answer” on the household income question were almost 3 times more likely than participants who reported their income to react negatively to the SOGI measures. Nonresponse was also higher for the income question (16%) than for the SOGI measures (2%). These results are supported by findings from other studies, which have shown that some populations find answering income questions to be more sensitive than SOGI questions.^40^ This item nonresponse may indicate that a subgroup of participants, albeit a small subgroup, have strong attitudes about what topics they are willing to answer and what should and should not be included in health research, a theme that also arose in some participants’ comments. In addition to the qualitative code “did not like the inclusion of SOGI,” “did not see connection with health” was considered a negative reaction; this code was assigned to comments that captured the sentiment that the CTS was stepping outside of its expected research areas by asking about SOGI. It is possible that participant expectations about relevant research areas were influenced by the CTS’s previous choices about which topics to include in the preceding surveys (Q1-Q5) that participants received.

In anticipation of some of these participant concerns about relevancy, the SOGI measures were introduced as a separate thematic topic toward the end of Q6 with the following introduction: “Research shows that individuals have unique health needs based on their sexual orientation and gender identity. We are asking the following questions to better understand those health experiences. If you would prefer not to answer these questions, please select ‘prefer not to answer’.” The qualitative feedback received from participants indicated that for some respondents, this explanation on the connection between SOGI and health was insufficient. Among CTS respondents with an ambiguous SOGI reaction, there was a subset who requested more clarity on why this information is important to research. Cohorts should consider the importance of participant-centered communication to convey why certain data are being collected.

There is growing agreement that health researchers should include SOGI measures in data collection tools.^41^ Previous studies have examined SGM perceptions of SOGI measures, assessed SOGI nonresponse rates among older adults, or evaluated whether sexual orientation measures affected study attrition within a pilot project.^24,42,43^ The CTS is one of the first established cohort studies to collect qualitative data on cohort participant reactions to SOGI measures introduced after study initiation and assess whether reactions to those measures varied by participant characteristics. Findings from our study suggest that including SOGI measures in a large-scale cohort helps support research on at-risk populations without affecting overall study attrition.^44^

## Limitations

This study did not directly ask participants to comment on the inclusion of the SOGI measures. These findings may underestimate participant sentiments regarding SOGI data collection. Because qualitative coding concluded before this project’s conception, the codebook was developed through inductive content analysis without consultation from members of the SGM community. One individual coded all 11 407 eligible feedback responses. Although particular attention was paid to SOGI-related comments, it is possible that some relevant comments were omitted. In this analysis, we used participants’ comments as indicators of participants’ sentiments. It is possible that the number of participants who commented on the SOGI measures is lower than the true number of participants who had feelings about the inclusion of SOGI on the questionnaire, and that some participants whose comments were coded as ambiguous in fact had stronger feelings about SOGI than was stated in their feedback. It is also possible that SOGI reactions among participants with missing data differed from reactions among the rest of the analytic population, but because missing data were less than 2%, we did not pursue multiple imputation or other adjustment. We included SOGI measures at the end of the questionnaire to minimize potential survey drop-off. Every CEC is different, and this decision seemed ideal for our study population at the time, but current SOGI data guidelines recommend collecting SOGI metrics with other demographic indicators.^9^ Web- and paper-based respondent demographics often differ^45,46^; because we did not include feedback questions on Q6paper, we could not assess whether SOGI reactions differed by questionnaire mode. The CTS includes current and retired California teachers or school administrators who had, at the time of Q6, answered up to 5 previous questionnaires. Based on the study location and existing relationship between the CTS and its participants, generalizability of results to other populations may be limited. Because the CTS is not a random sample of the population, these data should not be used to estimate the population prevalence of positive or negative reactions to SOGI questions among older adult women.

## Conclusion

This study provides important data on participant reactions to SOGI measures among a predominantly White, older population of volunteers who have been participating for over 20 years. Almost all participants responded to the SOGI questions, few participants commented on the SOGI measures, and among those who commented, the reactions were largely positive or ambiguous. Including SOGI measures in this CEC did not appear to affect overall study attrition. This study also shows the value of asking and systematically analyzing participant feedback about emerging research topics in large-scale ongoing studies. The low percentage of participants who reacted negatively to and opted out of future CTS research because of SOGI questions suggests that other studies should strongly consider collecting data on SOGI or other understudied communities.

## Supplementary Material

Supplementary material

Supplementary material is available at the *American Journal of Epidemiology* online.

## Figures and Tables

**Table 1.** Exemplar quotes, qualitative codes, and reaction groups: How were participants’ quotes coded and assigned to reaction groups?

Reaction groups	Rationale	Qualitative codes assigned	Exemplar quotes
Negative	These comments express a negative reaction in response to the sexual orientation and gender identity (SOGI) measures.	Did not like inclusion of SOGI	“All questions were very clear. It’s ashamed [sic] that the last few questions now include transgender, lesbian, gay etc. know it is a sign of the times, what a shame. Thank you”
			“I think those last few questions were ridiculous! Really? Gender choices?! I’m offended that a study I have been part of for so many years feels the need to be so PC as to bend to system so domineered by the current state of idiocy. You asked, and I always say it like I see it.”
	“Social engineering” was taken to refer to SOGI; these comments also express negative feelings.	Did not see connection to health	“Ask only health related questions—What does water source, gender identity and financial information have to do with health? Why does how long I have lived here matter to a health survey?”
			“I am not interested in participating in social engineering. I am willing to participate in a health survey. Please limit questions to that mission.”
Ambiguous	Comments categorized as “ambiguous” may express questions or commentary. They might also express positive and/or negative reactions but did not meet the criteria to be categorized positive or negative because they did not clearly refer to SOGI in conjunction with their reaction.	Modify SOGI questions	“Might be nice too, if there was a choice in the sexual orientation part to allow for considering yourself bisexual but having always behaved straight.”
			“I wonder about the ‘assigned’ at birth question about sex. Could it be phrased your biological sex?”
		Provide explanation	“well, I don’t know what intersex is and am thinking some people might not know exactly what transgender is either.”
			“Please provide an explanation to asking about one’s gender identity as well as sexual preference.”
		Inclusive	“It appears to be nondiscriminating.”
			“It’s pretty inclusive. Actually, it’s the best I’ve taken.”
		Too intrusive	“The questions are too intrusive. I realize you’re trying to do research, but I didn’t enjoy answering some of the questions.”
			“This Questionnaire was much too personal!!!”
		Reflects the times	“very thorough … .found it interesting that the questions were up to date regarding what is current in this generation”
			“Openness of the questions and still politically correct. Covered the questionable abspects [sic] of our society which is importations [sic] to consider in modern times. Well thought out”
		Respectful	“The questions were clear and concise. I appreciate the sensitivity and non-judgemental [sic] attitude of the survey.”
			“Choices covered all options, were respectful of all participants”
Positive	These comments express a positive reaction in response to the SOGI measures.	Liked inclusion of SOGI	“I was once married to a transgender person so I am pleased to see the recognition of this aspect of being. The questions were all non judgemental [sic].”
			“So happy about your method of identifying sexual orientation and gender identity. Go California Teachers Study!!!”

Abbreviation: SOGI, sexual orientation and gender identity.

**Table 2.** CTS participant characteristics at Q6 by reaction to SOGI measures (*n* = 19496).

	Total Q6 respondents	No reaction to SOGI^a^	Any reaction to SOGI^b^	*P* ^c^
Respondents	19 496 (100%)	19 123 (98%)	373 (2%)	
Age, years				
<65	5584 (29%)	5455 (29%)	129 (35%)	0.01
65-69	4058 (21%)	3985 (21%)	73 (20%)	
70-74	4670 (24%)	4585 (24%)	85 (23%)	
75-79	3083 (16%)	3020 (16%)	63 (17%)	
80+	2101 (11%)	2078 (11%)	23 (6%)	
Race^d^				
Non-White	1844 (10%)	1815 (10%)	29 (8%)	0.55^e^
Not reported	100 (1%)	99 (1%)	1 (0%)	
White	17 552 (90%)	17 209 (90%)	343 (92%)	
Sexual orientation				
Lesbian or gay	398 (2%)	386 (2%)	12 (3%)	ND^f^
Bisexual	(1%)**	(1%)**	(0%)**	
Straight	18 576 (95%)	18 231 (95%)	345 (93%)	
Other	23 (0%)	23 (0%)	0 (0%)	
Prefer not to answer	303 (2%)	292 (2%)	11 (3%)	
Do not know	(0%)**	(0%)**	(0%)**	
Missing	48 (0%)	48 (0%)	0 (0%)	
Gender identity				
Cisgender female	19 335 (99%)	18 976 (99%)	359 (96%)	ND^f^
Intersex	**	**	0 (0%)	
Gender expansive	**	**	0 (0%)	
Other	**	**	0 (0%)	
Prefer not to answer^g^	149 (1%)	135 (1%)	14 (4%)	
Marital status^h^				
Married/living with a partner	13 149 (67%)	12 892 (67%)	257 (69%)	0.21
Divorced	2532 (13%)	2483 (13%)	49 (13%)	
Separated	161 (1%)	154 (1%)	7 (2%)	
Widowed	2541 (13%)	2498 (13%)	43 (12%)	
Never married	1080 (6%)	1063 (6%)	17 (5%)	
Not reported/skipped	33 (0%)	33 (0%)	0 (0%)	
Annual household income^h^				
< $50 000	1548 (8%)	1518 (8%)	30 (8%)	0.16
$50 000-$74 999	2818 (15%)	2769 (15%)	49 (13%)	
$75 000-$99 999	3735 (19%)	3660 (19%)	75 (20%)	
$100 000-$149 999	4583 (24%)	4509 (24%)	74 (20%)	
$150 000-$199 999	2044 (11%)	2007 (11%)	37 (10%)	
$200 000+	1602 (8%)	1573 (8%)	29 (8%)	
Prefer not to answer	3111 (16%)	3032 (16%)	79 (21%)	
Not reported/skipped	55 (0%)	55 (0%)	0 (0.0%)	
Retirement status^h^				
Retired	14 317 (73%)	14 067 (74%)	250 (67%)	.01^e^
Not retired	4933 (25%)	4817 (25%)	116 (31%)	
Not reported/skipped	246 (1%)	239 (1%)	7 (2%)	

Abbreviations: CTS, California Teachers Study; SOGI, sexual orientation and gender identity.

a No reaction means the participant completed Q6 and did not provide a comment related to the inclusion of the SOGI measures.

b Any reaction means the participant completed Q6 and provided a comment about the SOGI measures (includes positive, negative, and ambiguous reactions).

c *P* value denotes differences between no reaction and any reaction groups.

d Data on race were collected at baseline. Not reported means the participant did not report race at baseline.

e Fisher exact test.

f Results that could not be generated due to small cell sizes are marked as “ND,” meaning not determined.

g These participants either marked prefer not to answer or skipped the question.

h These indicators were collected at Q6. Participants had the option to skip these questions on the web survey.

** To protect confidentiality and reduce the risk of identification, some frequencies (eg, cell counts less than 10) for potentially sensitive data have been replaced with asterisks.

**Table 3.** Characteristics of CTS participants by SOGI reaction type (*n* = 19 496)

	No reaction (*n* = 19 123)	Negative reaction (*n* = 151)	Ambiguous reaction (*n* = 129)	Positive reaction (*n* = 93)	Negative vs no reaction, *P*	Ambiguous vs no reaction, *P*	Positive vs no reaction, *P*
Age, years							
<65	5455 (29%)	54 (36%)	39 (30%)	36 (39%)	0.10^a^	0.75^a^	0.03^a^
65-69	3985 (21%)	27 (18%)	28 (22%)	18 (19%)			
70-74	4585 (24%)	33 (22%)	27 (21%)	25 (27%)			
75-79	3020 (16%)	28 (19%)	24 (19%)	11 (12%)			
80+	2078 (11%)	9 (6%)	11 (9%)	3 (3%)			
Race							
Non-White	1815 (9%)	6 (4%)	15 (12%)	8 (9%)	0.04^a^	0.68^a^	1.00^a^
Not reported	99 (1%)	**	**	**			
White	17 209 (90%)	144 (96%)	114 (88%)	85 (91%)			
Sexual orientation							
Lesbian or gay	386 (2%)	0 (0%)	**	**	ND^b^	ND^b^	ND^b^
Bisexual	(1%)**	**	**	**			
Straight	18 231 (95%)	140 (93%)	123 (95%)	82 (88%)			
Other	23 (0%)	0 (0%)	0 (0%)	0 (0%)			
Prefer not to answer	292 (2%)	**	**	0 (0%)			
Do not know	(0%)**	0 (0%)	0 (0%)	**			
Missing	48 (0%)	0 (0%)	0 (0%)	0 (0%)			
Gender identity							
Cisgender female	18 976 (99%)	139 (92%)	127 (98%)	93 (100%)	ND^b^	ND^b^	ND^b^
Intersex	**	0 (0%)	0 (0%)	0 (0%)			
Gender expansive	**	0 (0%)	0 (0%)	0 (0%)			
Other	**	0 (0%)	0 (0%)	0 (0%)			
Prefer not to answer	135 (1%)	12 (8%)	2 (2%)	0 (0%)			
Marital status							
Married/living with a partner	12 892 (67%)	110 (73%)	84 (65%)	63 (68%)	0.32	0.53	0.56
Divorced	2483 (13%)	16 (11%)	16 (12%)	17 (18%)			
Separated	154 (1%)	**	**	**			
Widowed	2498 (13%)	17 (11%)	18 (14%)	**			
Never married	1063 (6%)	**	**	**			
Not reported/skipped	33 (0%)	0 (0%)	0 (0%)	0 (0%)			
Annual household income							
< $50 000	1518 (8%)	11 (7%)	10 (8%)	9 (10%)	0.0004	0.52	0.33
$50 000-$74 999	2769 (14%)	16 (11%)	15 (12%)	18 (19%)			
$75 000-$99 999	3660 (19%)	30 (20%)	27 (21%)	18 (19%)			
$100 000-$149 999	4509 (24%)	26 (17%)	26 (20%)	22 (24%)			
$150 000-$199 999	2007 (11%)	10 (7%)	17 (13%)	10 (11%)			
$200 000+	1573 (8%)	12 (8%)	7 (5%)	10 (11%)			
Prefer not to answer	3032 (16%)	46 (30%)	27 (21%)	6 (6%)			
Not reported/skipped	55 (0%)	0 (0%)	0 (0%)	0 (0%)			
Retirement status							
Retired	14 067 (74%)	99 (66%)	90 (70%)	61 (66%)	0.07^a^	0.49^a^	0.07^a^
Not retired	4817 (25%)	50 (33%)	37 (29%)	29 (31%)			
Not reported/skipped	239 (1%)	2 (1%)	2 (2%)	3 (3%)			

Abbreviations: CTS, California Teachers Study; SOGI, sexual orientation and gender identity.

a Fisher exact test.

b Results that could not be generated due to small cell sizes are marked as “ND,” meaning not determined.

** To protect confidentiality and reduce the risk of identification, some frequencies (eg, cell counts less than 10) for potentially sensitive data have been replaced with asterisks.

**Table 4.** Associations between CTS participant characteristics and “any reaction” to SOGI measures^a,b^ (*n* = 19 119).

	Bivariables		Multivariables	
	OR	95% CI	OR	95% CI
Age, years^c,d^				
<65	1.40	1.06-1.85	1.43	1.08-1.89
65-74	1.10	0.84-1.44	1.11	0.85-1.45
75+	1.00	Ref	1.00	Ref
Race^d^				
Non-White	0.79	0.54-1.17	0.76	0.51-1.12
White	1.00	Ref	1.00	Ref
Marital status^c,e^				
Divorced/separated	1.05	0.78-1.41	1.08	0.80-1.45
Married/living with a partner	1.00	Ref	1.00	Ref
Never married/widowed	0.84	0.63-1.12	0.90	0.67-1.21
Annual household income^c,g^				
< $74 999	1.10	0.80-1.53	1.21	0.86-1.71
$75 000-$99 999	1.26	0.91-1.75	1.31	0.94-1.82
$100 000-$149 999	1.00	Ref	1.00	Ref
$150 000+	1.12	0.80-1.58	1.04	0.74-1.47
Prefer not to answer/not reported	1.60	1.16-2.21	1.72	1.24-2.39
Retirement status^f^				
Retired	1.00	Ref	1.00	Ref
Not retired	1.36	1.09-1.70	1.24	0.92-1.66

Abbreviations: CTS, California Teachers Study; OR, odds ratio; SOGI, sexual orientation and gender identity.

The reference group for this model is “no reaction.” The analytic population is participants who had any reaction (*n* = 365) or no reaction (*n* = 18 754).

a Any reaction means the participant completed Q6 and provided a comment about the SOGI measures (includes positive, negative, and ambiguous reactions).

b Participants were excluded from the bivariable and multivariable analyses if they had ANY missing/not reported responses. Sexual orientation and gender identity were not included in the bivariable and multivariable models due to small sizes in some groups.

c For the bivariable and multivariable analysis, age, marital status, and annual household income groupings were consolidated based on the distributions in Table 2 (eg, <65, 65-74, 65+).

d Model 1 independent variables included age group and race.

e Model 2 includes model 1 variables and marital status.

f Model 3 includes model 2 variables and retirement status.

g Model 4 includes model 3 variables and annual household income.

**Table 5.** Associations between CTS participant characteristics and a “negative reaction” to SOGI measures (*n* = 18 902).

	Bivariables		Multivariables	
	OR	95% CI	OR	95% CI
Age, years^a^				
<65	1.36	0.89-2.07	1.43	0.93-2.18
65-74	0.95	0.63-1.44	0.96	0.64-1.46
75+	1.00	Ref	1.00	Ref
Race^a^				
Non-White	0.40	0.18-0.91	0.38	0.17-0.87
White	1.00	Ref	1.00	Ref
Marital status^b^				
Divorced/separated	0.76	0.46-1.27	0.79	0.47-1.32
Married/living with a partner	1.00	Ref	1.00	Ref
Never married/widowed	0.73	0.46-1.15	0.76	0.47-1.22
Annual household income^d^				
< $74 999	1.09	0.63-1.89	1.32	0.74-2.35
$75 000-$99 999	1.43	0.83-2.44	1.54	0.90-2.65
$100 000-$149 999	1.00	Ref	1.00	Ref
$150 000+	1.11	0.63-1.97	0.97	0.54-1.73
Prefer not to answer/not reported	2.68	1.64-4.37	2.95	1.80-4.83
Retirement status^c^				
Retired	1.00	Ref	1.00	Ref
Not retired	1.49	1.06-2.10	1.38	0.88-2.17

Abbreviations: CTS, California Teachers Study; OR, odds ratio; SOGI, sexual orientation and gender identity.

The reference group for this model is “no reaction.” The analytic population is participants who had a negative reaction (*n* = 148) or no reaction (*n* = 18 754).

a Model 1 independent variables included age group and race.

b Model 2 includes model 1 variables and marital status.

c Model 3 includes model 2 variables and retirement status.

d Model 4 includes model 3 variables and annual household income.

**Table 6.** Associations between CTS participant characteristics and a “positive reaction” to SOGI measures (*n*= 18 844).

	Bivariables		Multivariables	
	OR	95% CI	OR	95% CI
Age, years^a^				
<65	2.28	1.22-4.26	2.30	1.23-4.32
65-74	1.83	1.00-3.35	1.84	1.00-3.36
75+	1.00	Ref	1.00	Ref
Race^a^				
Non-White	0.93	0.45-1.93	0.86	0.41-1.78
White	1.00	Ref	1.00	Ref
Marital status^b^				
Divorced/separated	1.46	0.86-2.48	1.55	0.91-2.63
Married/living with a partner	1.00	Ref	1.00	Ref
Never married/widowed	0.72	0.39-1.34	0.87	0.46-1.65
Annual household income^d^				
< $74 999	1.30	0.73-2.31	1.44	0.78-2.68
$75 000-$99 999	1.05	0.56-1.98	1.08	0.57-2.05
$100 000-$149 999	1.00	Ref	1.00	Ref
$150 000+	1.14	0.61-2.13	1.07	0.57-2.01
Prefer not to answer/not reported	0.42	0.17-1.03	0.47	0.19-1.17
Retirement status^c^				
Retired	1.00	Ref	1.00	Ref
Not retired	1.39	0.89-2.17	1.06	0.60-1.88

Abbreviations: CTS, California Teachers Study; OR, odd ratio; SOGI, sexual orientation and gender identity.

The reference group for this model is “no reaction.” The analytic population is participants who had a positive reaction (*n* = 90) or no reaction (*n* = 18 754).

a Model 1 independent variables included age group and race.

b Model 2 includes model 1 variables and marital status.

c Model 3 includes model 2 variables and retirement status.

d Model 4 includes model 3 variables and annual household income.

## Data Availability

All of the data associated with this publication and in the CTS are available for research use. The CTS welcomes all such inquiries and encourages individuals to visit https://www.calteachersstudy.org/for-researchers.
