# Supplementary material for: Participants’ reactions to the inclusion of sexual orientation and gender identity measures in an established large-scale cohort: the California Teachers Study
Source: Am J Epidemiol. Author manuscript; Available in PMC 2026 Feb 24. (PMC12930484; doi:10.1093/aje/kwaf074)
Supplement: Supplementary material [file NIHMS2141550-supplement-Supplementary_material.docx]

**Participants’ reactions to the inclusion of Sexual Orientation and Gender Identity (SOGI) measures in an established large-scale cohort: The California Teachers Study**

Kristen E. Savage, Christopher W. Wheldon, Emma S. Spielfogel, Brittany M. Charlton, Caroline A. Thompson, Christine N. Duffy, Maria Elena Martinez, James V. Lacey, Jr.

**Supplementary Material**

Table of Contents

[Appendix S1: Sexual Orientation and Gender Identity (SOGI) Feedback Qualitative Codebook 2](#_Toc194928474)

# Appendix S1: Sexual Orientation and Gender Identity (SOGI) Feedback Qualitative Codebook

| **Code** | **Definition** | **Used for Double Coding Selection** | **SOGI Relevance** | **Reaction Outcome** |
| --- | --- | --- | --- | --- |
| **Did not like inclusion of SOGI** | Participant explicitly said they did not like the inclusion of SOGI. | **Yes** | **Yes** | **Negative** |
| **Did not see connection to health** | Participant felt that the questionnaire included questions/topics that were unrelated to health; suggested the CTS stick to health. Suggested some questions or topics were "irrelevant" or "unnecessary". | **Yes** | **Yes** | **Negative** |
| **Inclusive** | Participant indicated that they liked the questionnaire seemed "inclusive" or "inclusive of their life". Unclear whether this means "inclusive", i.e. thorough/all encompassing, or if this means "inclusive" in the sense of diversity, inclusion, and equity. | **Yes** | **Yes** | **Ambivalent** |
| **Modify SOGI questions** | Provided suggestion that the SOGI questions be modified and/or may have provided suggestions for what changes could be made in terms of phrasing or answer choices. Also captures participants who asked that terms be defined, i.e. "define what intersex is." | **Yes** | **Yes** | **Ambivalent** |
| **Provide explanation about SOGI questions** | Participant asked probing questions about why SOGI questions were included and/or terms used in the SOGI questions. Captures questions/feedback that suggest these participants might have benefited from more explanation about the SOGI questions, including why they were included. | **Yes** | **Yes** | **Ambivalent** |
| **Reflects the times** | Participant felt that the topics and questions reflected the times, meaning that they appreciated that the questionnaire included "modern" topics like medicinal marijuana and sexual orientation & gender identity. | **Yes** | **Yes** | **Ambivalent** |
| **Respectful** | Participant said questionnaire was respectful, not intrusive, wasn't too personal, non-threatening, etc. | **Yes** | **Yes** | **Ambivalent** |
| **Too intrusive** | Participant found questionnaire invasive or intrusive. Captures sentiment where participant felt the questionnaire asked too many personal questions. | **Yes** | **Yes** | **Ambivalent** |
| **Liked inclusion of SOGI** | Participant liked that the questionnaire included questions on sexual orientation and/or gender identity. Captures participants who explicitly said "Includes SOGI questions" in response to "what did you like best about this questionnaire?" | **Yes** | **Yes** | **Positive** |
| **Remove income question** | Participant said questionnaire would be improved if we removed the income questions / participant did not like the inclusion of questions about income. | **Yes** | **No** | **N/A** |
| **Remove marijuana questions** | Participant felt that the CTS should remove the questions about medicinal marijuana and/or that these questions were out of scope for the study. Also captures participant who felt the inclusion of these questions signified that the CTS had "changed course" or was studying things they should not be. | **Yes** | **No** | **N/A** |
| **Remove organic food questions** | Participant felt that the CTS should remove the questions about organic food and/or that these questions were out of scope for the study. Also captures participant who felt the inclusion of these questions signified that the CTS had "changed course" or was studying things they should not be. | **Yes** | **No** | **N/A** |
| **Full Study Refusal** | Participant indicated in their response that they do not wish to continue in the California Teachers Study. This code does not capture the reason for full study refusal and therefore does not mean the refusal is due to SOGI, i.e., the participant could be refusing because they are too ill, no longer interested, etc. | **Yes** | **No** | **N/A** |
